# Mapping the sex determination locus in the hāpuku (*Polyprion oxygeneios*) using ddRAD sequencing

**DOI:** 10.1186/s12864-016-2773-4

**Published:** 2016-06-10

**Authors:** Jeremy K. Brown, John B. Taggart, Michaël Bekaert, Stefanie Wehner, Christos Palaiokostas, Alvin N. Setiawan, Jane E. Symonds, David J. Penman

**Affiliations:** Institute of Aquaculture, School of Natural Sciences, University of Stirling, Stirling, FK9 4LA Scotland, UK; The Roslin Institute, Royal (Dick) School of Veterinary Studies, University of Edinburgh, Easter Bush, Midlothian, EH25 9RG Scotland, UK; New Zealand National Institute of Water and Atmospheric Research, Bream Bay Aquaculture Park, Station Road, Ruakaka, 0151 New Zealand; Cawthron Institute, 98 Halifax Street East, Nelson, 7010 New Zealand

**Keywords:** Hapuku, Sex chromosome, Mariculture, RAD-seq, New Zealand groper

## Abstract

**Background:**

Hāpuku (*Polyprion oxygeneios*) is a member of the wreckfish family (Polyprionidae) and is highly regarded as a food fish. Although adults grow relatively slowly, juveniles exhibit low feed conversion ratios and can reach market size in 1–2 years, making *P. oxygeneios* a strong candidate for aquaculture. However, they can take over 5 years to reach sexual maturity in captivity and are not externally sexually dimorphic, complicating many aspects of broodstock management. Understanding the sex determination system of *P. oxygeneios* and developing accurate assays to assign genetic sex will contribute significantly towards its full-scale commercialisation.

**Results:**

DNA from parents and sexed offspring (*n* = 57) from a single family of captive bred *P. oxygeneios* was used as a template for double digestion Restriction-site Associated DNA (ddRAD) sequencing. Two libraries were constructed using *SbfI* – *Sph*I and *Sbf*I – *Nco*I restriction enzyme combinations, respectively. Two runs on an Illumina MiSeq platform generated 70,266,464 raw reads, identifying 19,669 RAD loci. A combined sex linkage map (1367 cM) was constructed based on 1575 Single Nucleotide Polymorphism (SNP) markers that resolved into 35 linkage groups. Sex-specific linkage maps were of similar size (1132 and 1168 cM for male and female maps respectively). A single major sex-determining locus, found to be heterogametic in males, was mapped to linkage group 14. Several markers were found to be in strong linkage disequilibrium with the sex-determining locus. Allele-specific PCR assays were developed for two of these markers, SphI6331 and SphI8298, and demonstrated to accurately differentiate sex in progeny within the same pedigree. Comparative genomic analyses indicated that many of the linkage groups within the *P. oxygeneios* map share a relatively high degree of homology with those published for the European seabass (*Dicentrarchus labrax*).

**Conclusion:**

*P. oxygeneios* has an XX/XY sex determination system. Evaluation of allele-specific PCR assays, based on the two SNP markers most closely associated with phenotypic sex, indicates that a simple molecular assay for sexing *P. oxygeneios* should be readily attainable. The high degree of synteny observed with *D. labrax* should aid further molecular genetic study and exploitation of hāpuku as a food fish.

**Electronic supplementary material:**

The online version of this article (doi:10.1186/s12864-016-2773-4) contains supplementary material, which is available to authorized users.

## Background

The hāpuku (*Polyprion oxygeneios*), or New Zealand groper, is a member of the wreckfish family (Polyprionidae). These large predatory fish range throughout the southern hemisphere and have been recorded in waters around New Zealand, southern Australia, southern America and a number of southern ocean archipelagos [1]. Although highly valued as a food and game fish, wild *P. oxygeneios* fisheries are relatively small scale and data on their exploitation status is limited [2–4]. *P. oxygeneios* juveniles are initially pelagic, moving to >100 m deep demersal habitats at around 3–4 years of age, where they can go on to reach 100 kg over an estimated 50–60 year lifespan [3, 4]. Although the species is generally slow growing, analysis of wild populations indicates that juveniles grow very rapidly during the first three years [3, 4]. These data are consistent with growth studies in captive juveniles of the closely related species, *Polyprion americanus* [5]. Moreover, growth studies undertaken by New Zealand National Institute of Water and Atmospheric Research (NIWA) have shown that *P. oxygeneios* can reach 1 kg in 12 months and 3 kg in 21–24 months, with commercially competitive feed conversion ratios (FCR) in the region of 1–2 [6, 7]. Market studies indicate that farmed portion size fish and fillets from larger fish are highly regarded in both local and international markets, making this species a strong candidate for aquaculture [7].

NIWA have an experimental captive breeding programme for wild-caught *P. oxygeneios* broodstock [8, 9] but maintaining a reliable supply of high quality fingerlings remains an obstacle to commercialisation of the species [7]. Wild *P. oxygeneios* are communal spawners and readily breed in captivity without the need for complex environmental or physiological interventions [8, 9]. However, their progeny take a relatively long time to reach sexual maturity and are not externally sexually dimorphic. Wild fish become sexually mature at an estimated 7–13 years, based on Western Australian [4] and New Zealand [3] stocks. Sexual maturation appears to occur slightly earlier in captive bred *P. oxygeneios*, such that male and female F1 stock can be differentiated at around 5 years of age using ultrasound imaging or evaluation of plasma vitellogenin or sex steroid levels [10]. All three of these methods only work reliably for adult captive broodstock in the run up to, and during the first half of, the August - December spawning season [10]. It would be advantageous to be able to sex *P. oxygeneios* at a much earlier age for broodstock management.

Analysis of sex ratios and gonad morphology in wild stocks indicates that *P. oxygeneios* is a primary gonochorist [4], and more than 10 years of captive breeding studies have yet to produced evidence to the contrary [11]. Aside from the observation that sex ratios in captive families are usually balanced (NIWA, unpublished data), very little is known about sex determination in *P. oxygeneios*. The mechanisms that determine sex in gonochoristic fish species are diverse; some rely purely on genetic determination, some on environmental cues and others on a combination of both [12, 13]. Although sex chromosomes are well differentiated in therian mammals and birds, which use male heterogamety (XX/XY) and female heterogamety (ZW/ZZ) to determine sex, respectively, the majority of fish species do not show sex chromosome differentiation. Sex chromosome pairs in fish species that do have XX/XY or ZW/ZZ sex-determining systems often show little or no heteromorphism [14], making sex identification problematic, even in species with known sex determination. Mapping the sex determination locus (or loci) using molecular markers would allow identification of sex-specific, or at least sex-linked, markers with which to develop molecular sexing methods.

The primary aim of the current study was to explore the genetics of sex-determination in hāpuku. Double digest Restriction-site Associated DNA (ddRAD) sequencing [15] was used to simultaneously identify and genotype a large number of novel Single Nucleotide Polymorphism (SNP) markers in the largest available captive-bred full-sib family for which phenotypic sex had been scored. The resultant genotypic data were used to construct the first genetic linkage maps for *P. oxygeneios,* and to search for potential sex-determining regions in this species. Two SNPs closely associated with phenotypic sex in the family were then developed as specific PCR assays and validated for their ability to differentiate genotypic sex. Additionally, the mapped ddRAD sequences were used to search for homologies with other ‘model’ fish species using comparative analyses.

## Methods

### Sample collection

All animal manipulations and handling were approved by the NIWA Animal Ethics Committee, in accordance with the national guidelines under the Animal Welfare Act 1999 of New Zealand. Wild-caught *P. oxygeneios* broodstock and F1 offspring were maintained at the NIWA aquaculture facility, Bream Bay, New Zealand [8]. Seven female and eight male broodstock were maintained in a 70 m^3^ communal spawning tank [9]. F1 juveniles from this batch spawning were fin clipped and tagged intraperitoneally with passive integrated transponders (PIT) [6]. Pedigrees were assigned using a proprietary pedigree analysis programme developed by AgResearch (New Zealand) whereby DNA profiles of the progeny were compared against all combinations of parents within the spawning tank [8]. F1 offspring from the largest represented full-sib family were sacrificed 432–439 days post-hatching (mean weight of 1454 ± 116 g) in November/December 2014. Gonads were fixed in 10 % neutral buffered formalin and routinely processed, embedded in paraffin wax, sectioned at 5 μm and stained with haematoxylin and eosin by New Zealand Veterinary Pathology [http://www.nzvp.co.nz]. F1 offspring were assigned phenotypic sex by histological examination of the gonads (Additional file 1: Figure S1). Fin clips from a single family (76 progeny; 38 males and 38 females, and two assigned parents) and 29 additional broodstock (14 males and 15 females) were collected into 100 % ethanol and shipped dehydrated at ambient temperature to the Institute of Aquaculture, University of Stirling, UK.

### Preparation of genomic DNA

After shipment fin clips were stored at 4 °C in 100 % ethanol prior to use. For DNA extraction fin clips were blotted dry and 10–15 mm^2^ samples transferred into nuclease-free 1.7 mL microfuge tubes. Samples were then incubated for 2 h at 55 °C with end-over-end mixing in 200 μl of CTAB buffer: 100 mM pH 8.0 trisHCl, 20 mM EDTA, 1.4 M NaCl, 55 mM hexadecyltrimethylammonium bromide, 0.2 % 2-mercaptoethanol (Sigma-Aldrich, Gillingham, UK); and 5 μL of proteinase K (10 mg/mL; Sigma-Aldrich). Once cooled to room temperature, 5 μL of Rnase A (2 mg/mL; Sigma-Aldrich) was added, the mixture then being incubated for a further 60 min at 37 °C with end-over-end mixing. Under a fume hood, samples were mixed well for 2 min with 110 μL of chloroform (Sigma-Aldrich) before centrifugation for 3 min at 12,000 *g*. Under a fume hood, 110 μL of the upper aqueous phase was transferred into a fresh nuclease-free 1.7 mL microfuge tube, to which 8.3 μL of 3 M sodium acetate pH 5.4 (Sigma-Aldrich) and 83 μL of isopropanol (Sigma-Aldrich) were added. DNA was precipitated by repeated sharp inversion of the samples followed by centrifugation at 12,000 *g* for 1 min. The supernatant was discarded and the pellet incubated in 1 mL of 70 % aqueous ethanol overnight at ambient temperature with end-over-end mixing. Samples were centrifuged at 21,000 *g* for 5 min and the supernatant discarded, the pellet was then air dried for 5 min at 55 °C and dissolved in 20 μL of 5 mM TrisHCl (pH 8) overnight at 4 °C. Total nucleic acid content and quality (260 nm/230 nm and 260 nm/280 nm ratios) were determined by spectrometry (Nanodrop; Thermo Scientific, Hemel Hempstead, UK) before adjusting nucleic acid concentrations to 100 ng/μL with 5 mM TrisHCl (pH 8). Double stranded DNA concentrations were then more accurately measured using a Qubit dsDNA Broad Range Assay Kit and Qubit Fluorometer (Invitrogen, Paisley, UK) and adjusted to 8 ng/μL with 5 mM TrisHCl (pH 8). As a final quality check 24 ng of each sample was then separated by electrophoresis, with 50 ng of Hind III digested lambda phage DNA as a size standard, in a 0.8 % agarose gel, 0.5× TAE buffer (20 mM Tris, 10 mM acetic acid, and 0.5 mM EDTA pH 8.0) containing 100 ng/mL of ethidium bromide (EtBr) to allow appraisal of genomic DNA quality and quantity prior to ddRAD library preparation. Genomic DNA of sufficiently high quality for generating ddRAD libraries was obtained from both parents and 58 of the 76 offspring supplied. Of the remaining 18 offspring, genomic DNA obtained from 16 individuals was deemed to be too degraded for inclusion in the libraries but adequate for use in subsequent PCR assays.

### ddRAD library preparation and sequencing

Following a modified version of the protocol described by Peterson et al. [15], two ddRAD libraries were prepared using different pairs of high fidelity restriction enzymes (REs) (New England Biolabs, UK; NEB); one using *Sbf*I (specific for the CCTGCA|GG motif) and *Sph*I (specific for the GCATG|C motif) and the other using *Sbf*I and *Nco*I (specific for the C|CATGG motif). The libraries were prepared consecutively, two weeks apart. For each library, samples from offspring (*n* = 58) were prepared in duplicate, while parental samples (*n* = 2) were performed in sextuplicate, to provide a three-fold overrepresentation of parental sequences. For each replicate, 3 μL of genomic DNA (24 ng) were individually digested at 37 °C for 45 min with 0.3 U of each RE pair in a final volume of 6 μL 1x CutSmart buffer (New England Biolabs). Samples were returned to ambient temperature for 5 min, and then incubated for 10 min, at ambient temperature, with 3 μL of unique combinations of RAD-specific P1 (6 nM) and P2 (72 nM) paired-end adapters [16] that included 5 or 7 bp barcodes (Additional file 2: Table S1). Barcoded adapters were designed such that adapter–genomic DNA combinations did not reconstitute RE sites, while residual RE activity limited concatemerisation of genomic fragments. The individual reactions were then incubated for 2 h at ambient temperature with 3 μL of ligase mastermix (1 mM rATP (Promega, Mannheim, Germany) and 2000 cohesive-end Units per μg of DNA of T4Ligase (New England Biolabs) in 1× CutSmart buffer). Ligase activity in each individual reaction was stopped by the addition of 30 μL PB buffer (pH indicator added) from a MinElute PCR purification kit (Qiagen, Manchester, UK) and the individual samples then pooled into one tube. The pooled reaction was pH adjusted with c. 2 μL 3 M sodium acetate pH 5.4 and sequentially loaded, in 550 μL aliquots, onto a single Minelute spin column and processed as per manufacturer’s instructions. The pooled library was eluted from the column in 2× 36 μL warmed EB buffer (60 °C) and stored on ice.

Size selection was performed by chilled electrophoresis in an EtBr-free 1.2 % agarose gel, 0.5× TAE buffer, loading the precooled sample (*c*. 68 μL of adapter ligated DNA mixed with 10 μL of 6× Ficol-based gel loading buffer into a single 25 mm wide lane flanked by lanes loaded with 710/416 base pair (bp) size markers. The gel was run at constant voltages of 45 V for 3 min, 60 V for 3 min and 90 V for around 70 min until the bromophenol blue dye front travelled approximately 3.5 cm from the origin. The central 2.2 cm wide by 4 cm long portion of the gel, that included the desired sample fractions (co-migrating roughly with the bromophenol blue marker dye), was excised with a scalpel and stored at 4 °C while the outer portion of the gel was post stained (0.5 μg/mL EtBr in 0.5× TAE buffer) for 10 min. Under UV transillumination, notches were cut in the outer portion of the gel to mark the location of the 710/416 bp size markers. The outer portion of the gel was washed in nuclease-free water and the central portion of the gel, containing the sample, replaced. Using the notches as guides, a horizontal slice was excised from the central section of the gel; ~6 mm thick, corresponding to a region containing c. 416 bp to c. 710 bp DNA fragments. The excised gel slice was processed through a spin-column (MinElute Gel Clean Up kit; Qiagen). The gel was dissolved in 3 volumes QG buffer at ambient temperature for 30 min with end-over-end mixing, the entire solution being sequentially loaded onto a single Minelute column and further processed following the manufacturer’s recommended protocol. The library template DNA was eluted from the column in two 35 μL volumes of warmed EB buffer (60 °C) and stored on ice (final volume ~65 μL).

Following optimisation of PCR conditions, to identify the minimum number of PCR cycles required to produce sufficient product for sequencing, a bulk amplification of each library (400 μL) was undertaken. For both libraries this large scale PCR comprised 32 μL library template, 9.6 μL combined 10 μM Illumina compatible P1 and P2 adapter specific primers [16], 200 μl Q5 Hot Start HF 2x Master Mix (New England Biolabs) and 160 μL nuclease-free water. After thorough mixing of the components, this mastermix was split into 32 times 12.5 μL separate reactions for PCR. Cycling conditions were: 98 °C for 1 min; then 12 cycles of 98 °C for 10 s, 65 °C for 15 s and 72 °C for 40 s; a final 72 °C for 3 min. Following PCR the 32 aliquots were recombined. To confirm successful amplification, 5 μL of the reaction was checked by electrophoresis (1.5 % agarose/TAE buffer gel; 100 ng/mL EtBr). The remainder was mixed with 3 volumes PB buffer, acidified with 2.5 μL of 3 M sodium acetate, and loaded in 550 μL aliquots onto a single MinElute PCR purification spin-column. The column was processed as per manufacturer’s instructions, the library being finally eluted in two 28 μL volumes of warmed EB buffer (60 °C). The library was further cleaned, to maximise removal of small fragments (< c. 200 bp), using an equal volume of Agencourt AMPure XP paramagnetic beads (Beckman Coulter, High Wycombe, UK) following the manufacturer’s instructions. The purified library was finally eluted in 18 μL EB buffer. For QC purposes 1 μL of purified library was visualised and sized by electrophoresis (1.5 % agarose gel; 0.5× TAE buffer; 100 ng/mL EtBr). DNA concentrations of both the purified library and the PCR template were measured by fluorimetry (Qubit dsDNA high sensitivity Assay Kit, Life Technologies, Paisley, UK). Finally, by taking into account the library size range and proportion of template present in the bulk PCR reaction, the purified library was diluted to 10 nM (amplicon equivalent) in EB buffer, 0.1 % Tween20, and stored at −20 °C until sequenced.

Sequencing was performed on the Illumina MiSeq platform (162 base paired end reads using a 300 base, v2 chemistry kit; Illumina, Cambridge, UK). Raw reads were processed using RTA 1.18.54 (Illumina). The raw sequence data from this study have been submitted to the EBI Sequence Read Archive (SRA) study PRJEB11817.

### Genotyping ddRAD alleles

The MiSeq generated reads were processed using a software pipeline designed specifically for RAD analysis, Stacks version 1.27 [17]. First, the *process_radtags* function was used to demultiplex the individual samples. During this step sequence reads with quality scores below 20, missing either restriction site or with ambiguous barcodes were discarded. Barcodes were removed and all sequences trimmed to be 135 bases long. For the purposes of this analysis paired-end reads were treated as separate loci, Read 2 sequences being appended to Read 1 sequence files. These sequences were assigned to RAD loci and genotypes using the ‘denovo_map.pl’ component of Stacks. The key parameter values employed in identifying RAD loci were; a minimum stack depth (m) of 10, a maximum of 2 mismatches allowed in a locus (M) in an individual and up to 1 mismatch between loci when building the catalog (n).

### Genetic linkage map construction

Taking a conservative approach, only data from RAD loci identified as containing one or two SNPs, i.e., those most likely to reflect true polymorphisms, were considered for analysis. These SNPs were further filtered, including only those identified in both parents and ≥75 % of offspring for analysis, and mapped using Lep-Map 2 [18]. Phenotypic sex was incorporated into the data set in both XX/XY and ZW/ZZ formats. Markers deviating from expected Mendelian segregation (*P* < 0.01) were excluded from further analysis using the option “dataTolerance = 0.01” within the *Filtering* module. Lep-Map 2 was used to generate male, female and consensus maps during the execution of all subsequent modules by including the commands; “informativeMask = 13” for the male map, “informativeMask = 23” for the female map and “informativeMask = 123” for the consensus map. Linkage groups were generated using the modules *SeparateChromosomes* and *JoinSingles* with options “sizeLimit = 10” and “lodLimit = 8” to limit linkage group generated to ≥10 markers with a minimum logarithm of the odds (LOD) value of 8. The module “OrderMarkers” with options “useKosambi = 1” and “maxDistance = 50” was used to determine the order and distance between markers in centiMorgans (cM) using the Kosambi mapping function [19], splitting linkage groups that had gaps of ≥50 cM between markers. The option “sexAveraged = 1” was included during execution of *OrderMarkers* when constructing the consensus map. Similar parameters were also used to construct a consensus map using the R/OneMap package [20] release 2.0-4. The genetic maps were drawn and aligned using Genetic-Mapper v0.6 [21].

### Association analysis

Association analysis was performed using the R/SNPassoc package [22] release 1.9-2 to test for associations between SNP genotypes and phenotypic sex using the function *WGassociation* with options “model = "codominant”. Bonferroni correction was used to counteract the problem of multiple comparisons when determining the significance of observed results.

### Allele-specific PCR assays

Allele-specific PCR primers [23] were designed for the two SNPs that were most closely associated with phenotypic sex using Primer Select software (DNASTAR Inc. Madison, USA). Specificity was achieved by designing two forward primers, one for each allele, with the SNP at the 3’ end preceded by a mismatched nucleotide; and a single common reverse primer. Both assays performed optimally with a 60 °C annealing step. Allele-specific PCR was performed on a subsample of RAD-screened offspring (for assay validation: *n* = 10) and on other offspring from the same pedigree (*n* = 16), which were not included in the ddRAD libraries due to their relatively poor genomic DNA integrity. Allele-specific PCR was also performed on samples from unrelated stock to estimate SNP allelic frequencies and association with phenotypic sex in the wider population (*n* = 29). Amplicons were separated by electrophoresis (2 % agarose gel, 0.5× TAE buffer, containing 100 ng/mL ethidium bromide) with repeat loading of pairs of allele-specific amplicons at 5 min intervals into the same wells, to resolve double-banded phenotypes for heterozygous and single-banded phenotypes of different mobility for both homozygous phenotypes.

### Comparative genomics

Consensus marker sequences were used as a query for homology searches against available fish genomes with BLASTn v.2.2.28+ [24]. Reference sequences of *Astyanax mexicanus* (AstMex102), *Danio rerio* (GRCz10), *Dicentrarchus labrax* (dicLab1), *Gadus morhua* (gadMor1), *Gasterosteus aculeatus* (BROAD_S1), *Latimeria chalumnae* (LatCha1), *Lepisosteus oculatus* (LepOcu1), *Oreochromis niloticus* (Orenil1.1), *Oryzias latipes* (HdrR), *Petromyzon marinus* (Pmarinus_7.0), *Poecilia formosa* (Poecilia_formosa-5.1.2), *Salmo salar* (ICSASG_v2), *Takifugu rubripes* (FUGU 4.0), *Tetraodon nigroviridis* (TETRAODON8.0) and *Xiphophorus maculatus* (Xipmac4.4.2) were downloaded from the NCBI Genome Assembly database.

For the further analyses only RAD loci present in the *P. oxygeneios* linkage map and their putative homologs in the other fish genomes were taken into account. These RAD loci were repeat masked and the best hit for each RAD locus was deemed to be homologous if it covered at least 50 % of the sequence with at least 50 % identity. Detected homologies within *D. labrax* were visualized with Circos [25] release 0.68.

## Results

### ddRAD sequencing

**Fig. 1:**
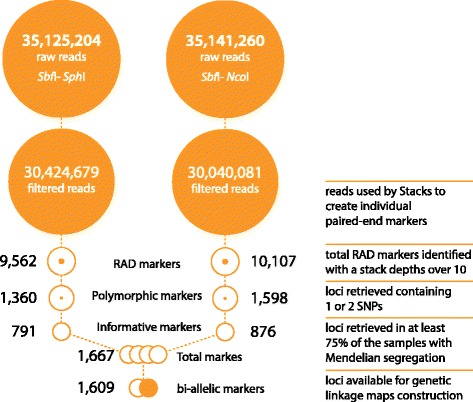
Pictorial summary of the sequencing pipeline. Total numbers of reads obtained from each library are shown; raw and filtered for low quality sequences (quality score < 20) and reads with missing or ambiguous barcodes, followed by the number of polymorphic ddRAD markers identified by Stacks, and the number of informative markers that were subsequently used to generate genetic linkage maps and in genome-wide association studies

Total numbers of raw reads and filtered reads obtained from each library using the Illumina MiSeq platform are shown in Fig. 1. Overall the performance and yield of both RAD loci and informative SNPs was broadly similar for the two libraries. Consistent with their deliberate over-representation in both libraries, in order to more robustly identify true SNPs, 2 to 3 fold more reads were obtained for the parents (Additional file 2: Table S1). After removal of low quality sequences and reads with missing or ambiguous barcodes, 86.6 % of reads in the *Sbf*I – *Sph*I library and 85.5 % of reads in the *Sbf*I – *Nco*I library were retained. One of the offspring, #86, was approximately 10-fold underrepresented cf. the others in both libraries and was excluded from subsequent analysis on this basis (Additional file 2: Table S1). *De novo* building and genotyping of loci with Stacks identified a total of more than 10,000 RAD loci in each library. Of these, 1360 in the *Sbf*I – *Sph*I library and 1598 in the *Sbf*I – *Nco*I library contained 1–2 SNPs. A combined total of 1667 markers were informative in one or both sexes, present in at least 75 % of the offspring and adhered to expected Mendelian segregation ratios (*χ*^2^*p*-value > 0.01). All of these markers were subsequently used to construct genetic linkage maps (Additional file 3: Table S2). The 1609 bi-allelic markers present in this panel were also used in association analysis.

### Genetic linkage maps

**Fig. 2:**
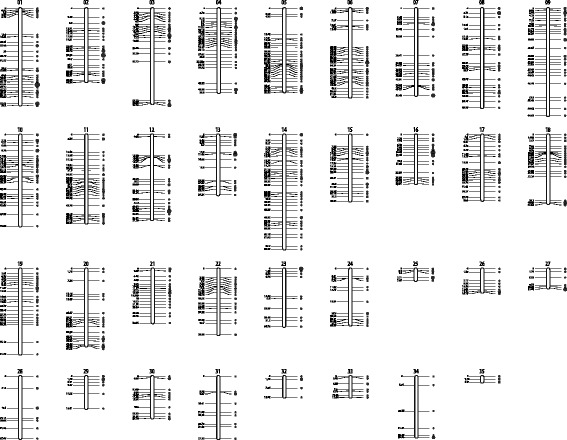
*P. oxygeneios* Genetic linkage map. The consensus map generated using Lep-Map 2 software contained a total of 1575 informative SNP markers arranged into 35 linkage groups. The positions on the left side of the chromosomes are in centiMorgans. The diameters of the filled circles on the right hand side are proportionate to the number of markers at those positions. A more detailed map is provided in Additional file 3: Table S2

**Fig. 3:**
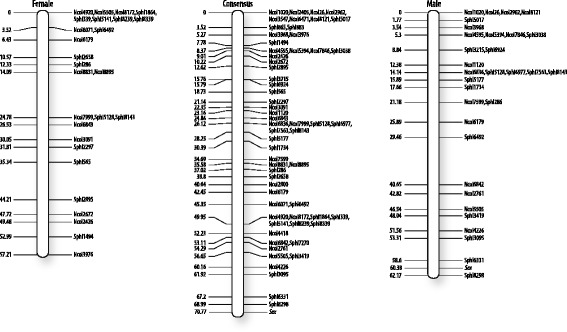
Putative sex chromosome *of P. oxygeneios*. Detailed maps of linkage group 14 from the consensus, male and female maps are shown, generated using Lep-Map 2 software. The positions on the left side of the chromosomes are in cM. The annotations on the right hand side identify the specific polymorphic SNP markers mapped to those positions

Consensus, female and male genetic linkage groups were constructed for *P. oxygeneios* using Lep-Map 2 (Table 1). The consensus map was constructed using 1667 informative markers, 1575 of which mapped to 35 linkage groups with ≥10 markers, spanning a total distance of 1366.4 cM (Fig. 2, Additional file 4: Table S3). Similar results were obtained using R/OneMap (data not shown). The 1168 cM female-specific map was constructed using 819 markers that were informative in the female (Additional file 5: Figure S2, Additional file 6: Table S4). Similarly, the 1132 cM male-specific map was constructed using 878 markers that were informative in the male (Additional file 7: Figure S3). In both cases, linkage group identities were matched to those of the consensus map (Fig. 3, Additional file 8: Table S5). When phenotypic sex of the offspring was coded as XX/XY genotypes and included in the linkage analysis, ‘sex’ localised to the end of linkage group 14 in both the consensus and male maps but was absent from the female-specific map (Fig. 3). When scored as ZW/WW genotypes, ‘sex’ remained unlinked in all three map analyses.

**Table 1:** *P. oxygeneios* linkage maps

Linkage group	Consensus		Female		Male	
	Markers	Size (cM)	Markers	Size (cM)	Markers	Size (cM)
1	90	58.30	63	54.7	44	60.39
2	78	44.24	41	49.55	50	21.08
3	75	57.53	46	32.34	39	97.38
4	73	50.80	39	65.7	49	48.58
5	75	50.35	43	46.14	35	21.09
6	70	53.60	37	46.13	47	87.47
7	65	52.49	48	88.22	27	34.15
8	65	59.89	40	60.08	45	62.07
9	66	64.62	23	45.99	45	64.04
10	69	55.08	28	42.49	44	40.56
11	66	53.34	23	19.45	34	54.75
12	58	51.29	37	50.72	28	46.94
13	57	36.26	25	56.26	43	19.34
14	62	70.77	27	57.21	33	62.17
15	57	40.05	33	45.64	26	41
16	55	29.51	22	26.68	33	19.32
17	47	39.60	17	40.31	38	22.84
18	47	41.52	12	43.12	33	31.64
19	53	51.49	27	47.33	27	52.28
20	42	46.55	13	32.59	37	35.53
21	43	32.64	10	8.01	35	33.42
22	30	39.69	10	26.42	17	26.43
23	24	34.78	18	38.11	15	49.47
24	23	33.99	23	28.25	-	-
25	21	7.07	10	0	12	12.33
26	19	14.19	17	10.55	-	-
27	19	11.61	10	1.76	11	17.64
28	19	37.49	16	31.47	-	-
29	18	19.41	-	-	18	19.41
30	23	25.18	15	4	13	50.55
31	17	37.55	17	21.15	-	-
32	13	12.62	13	12.62	-	-
33	13	12.64	-	-	-	-
34	13	36.74	10	33.18	-	-
35	10	3.51	10	1.76	-	-
Total	1575	1366.39	819	1167.93	878	1131.87

### Association analysis

**Fig. 4:**
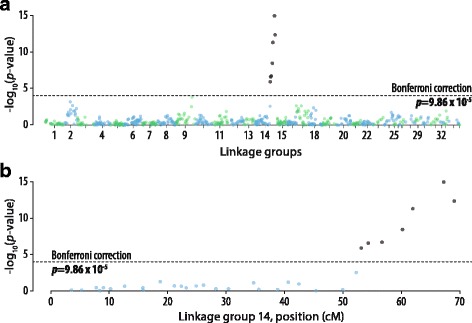
Association analysis of informative SNPs and phenotypic sex. **a** A plot of the association of 543 unique positions (identified by 1522 informative SNPs) with phenotypic sex, scaled along the x-axis to their position (cM) in the *P. oxygeneios* genetic linkage map. **b** A higher resolution representation of the portion of linkage group 14 adjacent to the putative sex-determining locus, showing SNP marker identities. Dashed line represents Bonferroni corrected significance level (*P* < 0.05), based on 543 unique observations

Association analysis identified seven markers that appear to be significantly associated with phenotypic sex in *P. oxygeneios* (Fig. 4). When ordered using the *P. oxygeneios* consensus linkage map, all seven markers located to one end of linkage group 14, adjacent to male phenotypic sex (Fig. 4b). The two markers most closely associated with male phenotypic sex in linkage group 14 (Fig. 3: *Sph*I6331 and *Sph*I8298) also showed the closest association with phenotypic sex association analysis (Fig. 4b: *Sph*I6331 *P* = 1.4 × 10^−15^ and *Sph*I8298 *P* = 4.5 × 10^−13^).

### Sex prediction using allele-specific PCR assays

Allele-specific PCR primers were designed for markers *Sph*I6331 and *Sph*I8298 (Additional file 9: Table S6). DNA from known RAD-genotyped samples (5 males and 5 female offspring), together with DNA from 16 other phenotypically sexed offspring were screened, *i.e.,* those samples for which extracted DNA was considered to be too degraded for inclusion in the ddRAD libraries. For *Sph*I6331, the sire was heterozygous (AT) and the dam was homozygous (TT); 25 progeny conformed to this pattern, while one female was heterozygous (Additional file 10: Figure S4). For *Sph*I8298, the sire was heterozygous (CT) and the dam was homozygous (TT): all male progeny were TT and all female progeny were CT (Additional file 10: Figure S4). The A allele of *Sph*I6331 thus appears to be associated with the Y sex-determining allele in this family, while for *Sph*I8298 the paternal T allele appears to be associated with the Y (and the paternal C allele with the X). For the addition 29 unrelated broodstock, observed heterozygosity was relatively high at both loci (*Sph*I6331 = 0.38 and *Sph*I8298 = 0.34) but phenotypic sex within this group was not associated with genotype at either locus.

### Comparative genomics

**Fig. 5:**
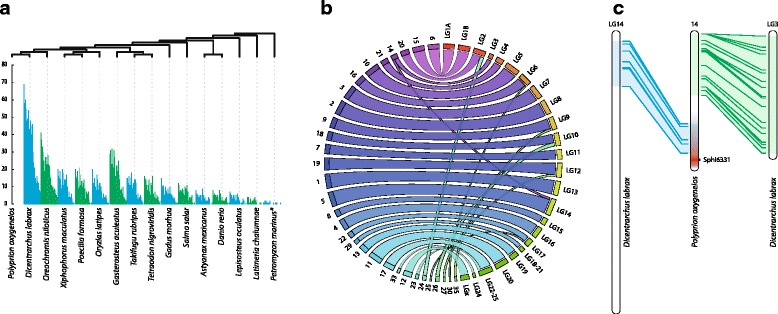
Comparative genomics analysis. **a** The percentage of *P. oxygeneios* ddRAD sequences per linkage group that showed homology with sequences from other fish genomes, listed on the x-axis; *P. marinus: outgoup. The phylogenetic tree provided above the graph is based on recent classifications [30–32]. **b** Schematic of homology between proposed linkage groups (1–35) and published *D. labrax* linkage groups. Only blocks of 2 markers or more are illustrated; The complete comparative analysis for all species is summarised in Additional file 11: Table S7. **c** Graphic identifying syntenic regions between *P. oxygeneios* LG 14 and *D. labrax* linkage groups

Homology searches were performed against other fish genomes using the RAD loci included in the *P. oxygeneios* linkage map (Fig. 5, Additional file 11: Table S7). This was undertaken primarily in order to validate the map build but also to establish the extent to which existing fish genome resources could prove informative for future studies of this species.

The fewest ‘homologous’ loci were found in the comparison with *L. oculatus* and *L. chalumnae*, the only non-teleost species in the set of genomes explored (*P. marinus* were used as an outgoups in Fig. 5a and Table 2). Homologous sequences to more than 200 *P. oxygeneios* ddRAD loci were found in *O. niloticus*, *G. aculeatus, X. maculatus*, *P. formosa*, *T. rubripes*, *O. latipes* and *T. nigroviridis* (Fig. 5a, Table 2). Of all the species examined, *D. labrax* exhibited by far the highest degree of homology with *P. oxygeneios*. Approximately 75 % of the analysed sequences in *P. oxygeneios* have homologous sequences within the *D. labrax* genome (Fig. 5). *P. oxygeneios* linkage groups also appear to show a high degree of homology with *D. labrax* linkage groups (Fig. 5b), as every linkage group of *P. oxygeneois* is clearly linked to one linkage group in the *D. labrax* with exception of LG 14. Loci located in LG 14 of *P. oxygeneois* are mainly split between linkage groups 3 and 14 in *D. labrax* with marker order within each of these two *D. labrax* groups being highly conserved with P. *. oxygeneois* (Fig. 5c). Overall, there is a strong correlation of each hāpuku linkage group to a single LG not only in *D. labrax* but also in *O. niloticus* and *G. aculeatus,* where marker order also appears to be generally conserved (Additional file 11: Table S7). These observations support the contention that the hāpuku linkage map is reliable and robust.

**Table 2:** Comparative genomics of *P. oxygeneios*

Species	Common name	Number of homologous loci
*Dicentrarchus labrax*	European seabass	1182
*Oreochromis niloticus*	Nile tilapia	616
*Gasterosteus aculeatus*	Three-spined stickleback	525
*Xiphophorus maculatus*	Southern platyfish	323
*Poecilia formosa*	Amazon molly	310
*Takifugu rubripes*	Japanese puffer	279
*Oryzias latipes*	Medaka	251
*Tetraodon nigroviridis*	Green spotted puffer	247
*Salmo salar*	Atlantic salmon	185
*Gadus morhua*	Atlantic Cod	180
*Astyanax mexicanus*	Mexican tetra	111
*Danio rerio*	Zebrafish	108
*Lepisosteus oculatus*	Spotted gar	84
*Latimeria chalumnae*	Coelacanth	39
*Petromyzon marinus* ^a^	Lamprey	17

Number of mapped RAD loci in Hāpuku that shared homology with other fish genomes. ^a^ *P. marinus*: outgoup

## Discussion

In the absence of pre-existing sequence or karyotype data, sequencing of two independent ddRAD libraries from a single full-sib family on an Illumina MiSeq has shown that *P. oxygeneios* has an XX/XY sex determination system and allowed identification of several SNPs that are associated with phenotypic sex. In a departure from the methodology described by Peterson et al. [15], individual samples were pooled immediately after ligation with barcodes and prior to PCR amplification during preparation of both *P. oxygeneios* ddRAD libraries. This approach greatly reduces both the time required to prepare libraries and the potential for fragment size variation caused by size-selecting smaller groups of individuals prior to pooling into a single library for sequencing. The relative consistency in the number of reads obtained among individuals in both libraries (Additional file 2: Table S1) validates this approach. The use of two different RE pairs, which shared a common eight base recognition RE, *Sbf*I, but used different 6 base recognition REs *Sph*I and *Nco*l to generate ddRAD fragments, resulted in the identification of approximately twice as many mappable RAD markers as either pair used in isolation. Only 43 mapped loci were common to both libraries. In total, this approach produced 1667 SNPs that were informative for the purposes of genetic map construction, 1609 of which were bi-allelic and available for association analysis.

In the absence of karyotype information for *P. oxygeneios*, a relatively high minimum LOD score of 8 was used to construct the genetic linkage map, resulting in 35 linkage groups being identified. This is perhaps higher than the number of chromosomes that one might expect to observe in *P. oxygeneios*, based on its relative homology with *D. labrax*, which has 24 pairs [26]. It was considered preferable to split linkage groups where there were large gaps between markers, rather than join potentially independent linkage groups. A drawback associated with this approach is that if phenotypic sex was not closely linked with any SNPs identified in the library then it might be excluded from the map completely. Fortunately, two of the markers with SNPs that were informative in the male offspring, *Sph*I6331 and *Sph*I8298, were located within 2 cM of phenotypic sex on linkage group 14. The association analysis and subsequent allele-specific PCR screening of the offspring that were not included in the libraries strongly supported these associations. Taken collectively, these findings indicate that *P. oxygeneios* has an XX/XY sex determination system, in which the major sex determining region is located in linkage group 14 and that SNPs in markers *Sph*I6331 and *Sph*I8298 are in strong linkage disequilibrium with the gene(s) within this locus.

Given the wide range of sex determination systems that operate in fish [13, 14], the discovery that sex determination in *P. oxygeneios* appears to be monogenic is encouraging with respect to developing sex determination assays. The results of the allele specific PCR assays for the two most closely associated loci, *Sph*I6331 and *Sph*I8298, appear to bear this out. However, the alleles at these loci are not sex-specific. This is illustrated by the fact that while the sire is heterozygous at *Sph*I8298, the male offspring in the family were nearly all homozygous at this locus and thus the *Sph*I8298 genotype would not be informative in the next generation. This is consistent with *Sph*I8298 being located near the sex-determining locus, and likely to segregate with sex, but playing no direct role in sex determination. Although *Sph*I6331 was heterozygous in both sire and male offspring it is located at around the same distance (~2 cM) from the sex determination locus as *Sph*I8298. Additionally, the allele-specific PCR assay for *Sph*I6331 was not 100 % accurate at predicting sex, which is consistent with the ddRAD library results and probably reflects meiotic crossover in the 2 cM interval between this marker and the sex determination locus. However, a “false” positive band (corresponding to A) in what was expected to be a homozygous TT female could also be an artefact of the allele-specific primers [23] initiating the PCR at the nucleotide immediately after the mismatch. This could account for the slightly smaller amplicon observed in this sample and the faint band at the same size observed in one of the other females. This may be resolved by further optimisation of the PCR conditions or adoption of potentially more stringent allele specific assays, such as Kompetitive Allele Specific PCR [27]. Given that heterozygous individuals appear to be relatively common in the wider population (0.38–0.41), it seems likely that these markers will be useful outside the current family where parental genotypic information is available and communal spawning groups can be structured to ensure that either, or both, markers are informative in the offspring. However, it may be necessary to extend the RAD sequencing studies to additional, larger, families or outbred populations to obtain the resolution required to identify markers that are more closely sex-linked than those identified in the current study. Ultimately, the genetic map generated in the current study will make possible mapping and association studies for any trait of interest with a view to long-term genetic improvement of captive *P. oxygeneios* lines [28].

Homology searches, comparing the RAD loci of *P. oxygeneios* with other fish genomes, demonstrate that a high degree of sequence homology exists with the European sea bass, *D. labrax* at chromosomal level, and to a lesser extent with *O. niloticus* and *G. aculeatus.* Marker order within linkage groups is also conserved, suggesting that the *D. labrax* genome, in particular, should be a valuable resource to aid future *P. oxygeneios* genetic studies. In contrast to what appears to be a relatively simply monogenic sex determination system in *P. oxygeneios*, sex determination in *D. labrax* is regulated through a combination of polygenic and environmental factors [26, 29]. Interestingly, *P. oxygeneios* linkage group 14 is unusual in that its markers show sequence homology with sequences in multiple chromosomes in *D. labrax*.

## Conclusions

The current study has produced the first-ever genetic linkage map of *P. oxygeneios*, using ddRAD sequencing. The map consists of 1575 loci in 35 linkage groups and supports the location of a single sex-determining region near one end of linkage group 14 in the male-specific and consensus maps. This is strongly indicative that *P. oxygeneios* has an XX/XY sex determination system. This study has also identified several novel markers that are in strong linkage disequilibrium with the gene(s) within the sex-determining region, albeit within the single family analysed. Allele-specific PCRs were developed for two of these markers, *Sph*I6331 and *Sph*I8298, and demonstrated to be able to accurately predict male and female offspring within the same family. Further analysis of unrelated individuals showed no population-wide association with phenotypic sex, but showed that heterozygosity is relatively common at both loci in the wider population, indicating that they are likely to be useful markers when sexing individuals within a pedigree situation. Finally, comparative genomic analyses indicate that many of the linkage groups within the *P. oxygeneios* map share a relatively high degree of homology with those published for *D. labrax* and that marker order within linkage groups appears highly conserved. It is hoped that these findings will lead to the development of more robust assays for genotypic sex, allowing a much greater degree of control of sex ratios in brood stock management, ongrowing and, ultimately, lead to genetic improvement of this promising new aquaculture species.

## Abbreviations

cM, centiMorgans; ddRAD, double digest Restriction-site Associated DNA; FCR, feed conversion ratio; LOD, logarithm of the odds; NGS, next generation sequencing; NIWA, New Zealand National Institute of Water and Atmospheric Research; PIT, passive integrated transponders; RAD, Restriction-site Associated DNA; RE, restriction enzyme; SNP, Single Nucleotide Polymorphism.

## Additional files

Additional file 1: Figure S1. Phenotypic sexing. Representative histological presentation of female (A and B) and male (C and D) *P. oxygeneios* gonads at 432–439 days post hatching. Scale bar represents 50 μm. (TIF 31728 kb) Additional file 2: Table S1. Detailed information for each sample used. Sample ID, Barcodes, Read recovered etc. (CSV 12 kb) Additional file 3: Table S2. Full genotypes for mapping family. (CSV 301 kb) Additional file 4: Table S3. Consensus genetic map (positions and RAD locus sequences). (CSV 235 kb) Additional file 5: Figure S2. Female only map of all SNP markers. The positions on the left side of chromosomes are the distance in centi Morgan (cM), Detailed data is provided in Table S2. (PDF 849 kb) Additional file 6: Table S4. Female only genetic map (positions and RAD locus sequences). (CSV 122 kb) Additional file 7: Figure S3. Male only map of all SNP markers. The positions on the left side of chromosomes are the distance in centi Morgan (cM), Detailed data is provided in Table S2. (PDF 850 kb) Additional file 8: Table S5. Male only genetic map (positions and RAD locus sequences). (CSV 130 kb) Additional file 9: Table S6. Primer and sequence information for allele-specific SNP assays. (CSV 597 bytes) Additional file 10: Figure S4. Gel images of SNP assays for SphI8298 and SphI6331. (PDF 209 kb) Additional file 11: Table S7. Synteny comparisons. Location of each marker retrieved in tested genomes. For each marker, the chromosome ID, start and end location of the best BLASTn hit is reported. (CSV 138 kb)
