# Supplementary figures and images for: Mapping the sex determination locus in the hāpuku (Polyprion oxygeneios) using ddRAD sequencing
Source: BMC Genomics. 2016 Jun 10;17:448. doi: 10.1186/s12864-016-2773-4 (PMC4902995; doi:10.1186/s12864-016-2773-4)

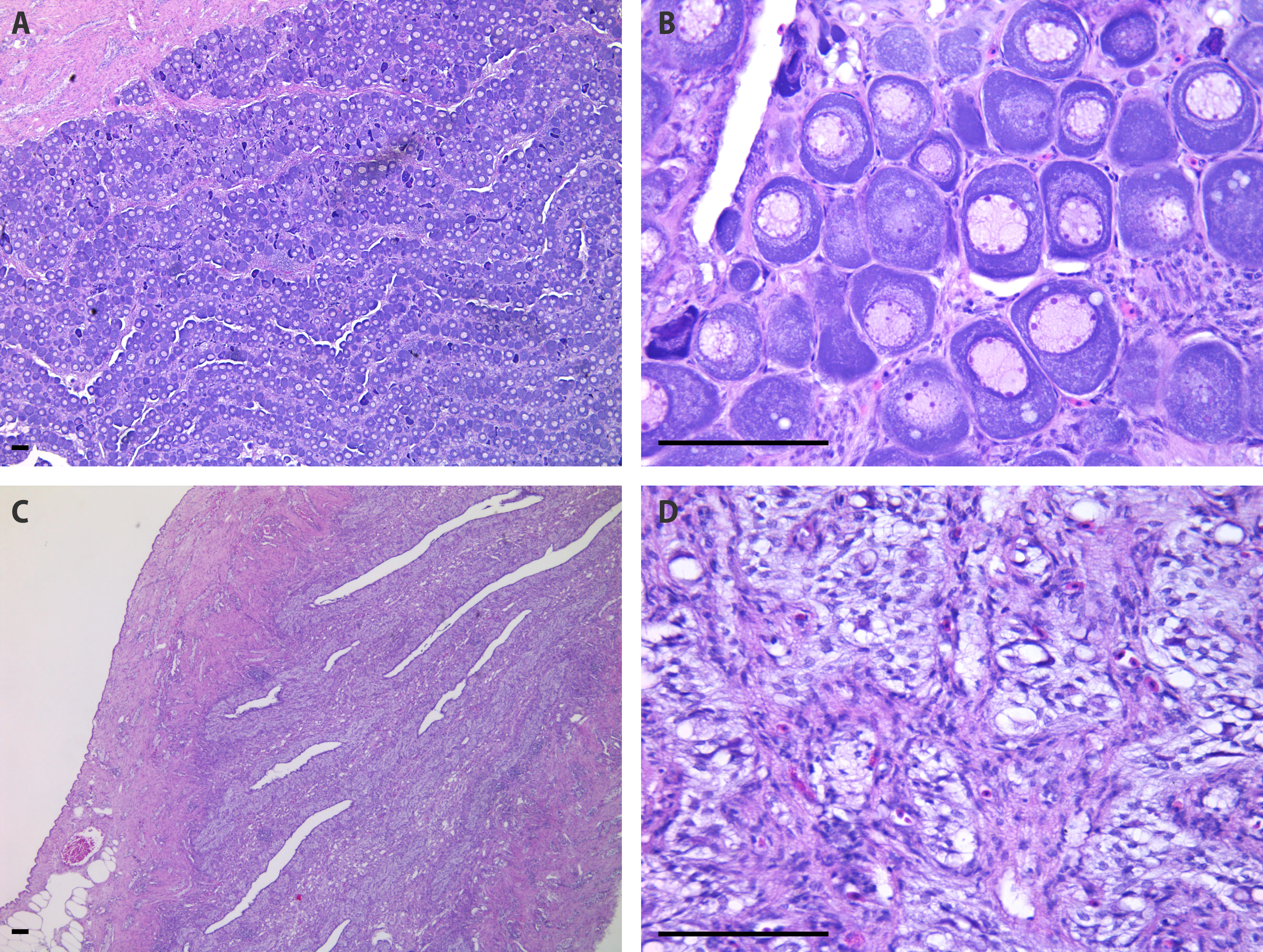

Supplement: Additional file 1: Figure S1. — Phenotypic sexing. Representative histological presentation of female (A and B) and male (C and D) P. oxygeneios gonads at 432–439 days post hatching. Scale bar represents 50 μm. (TIF 31728 kb) [file 12864_2016_2773_MOESM1_ESM.tif]

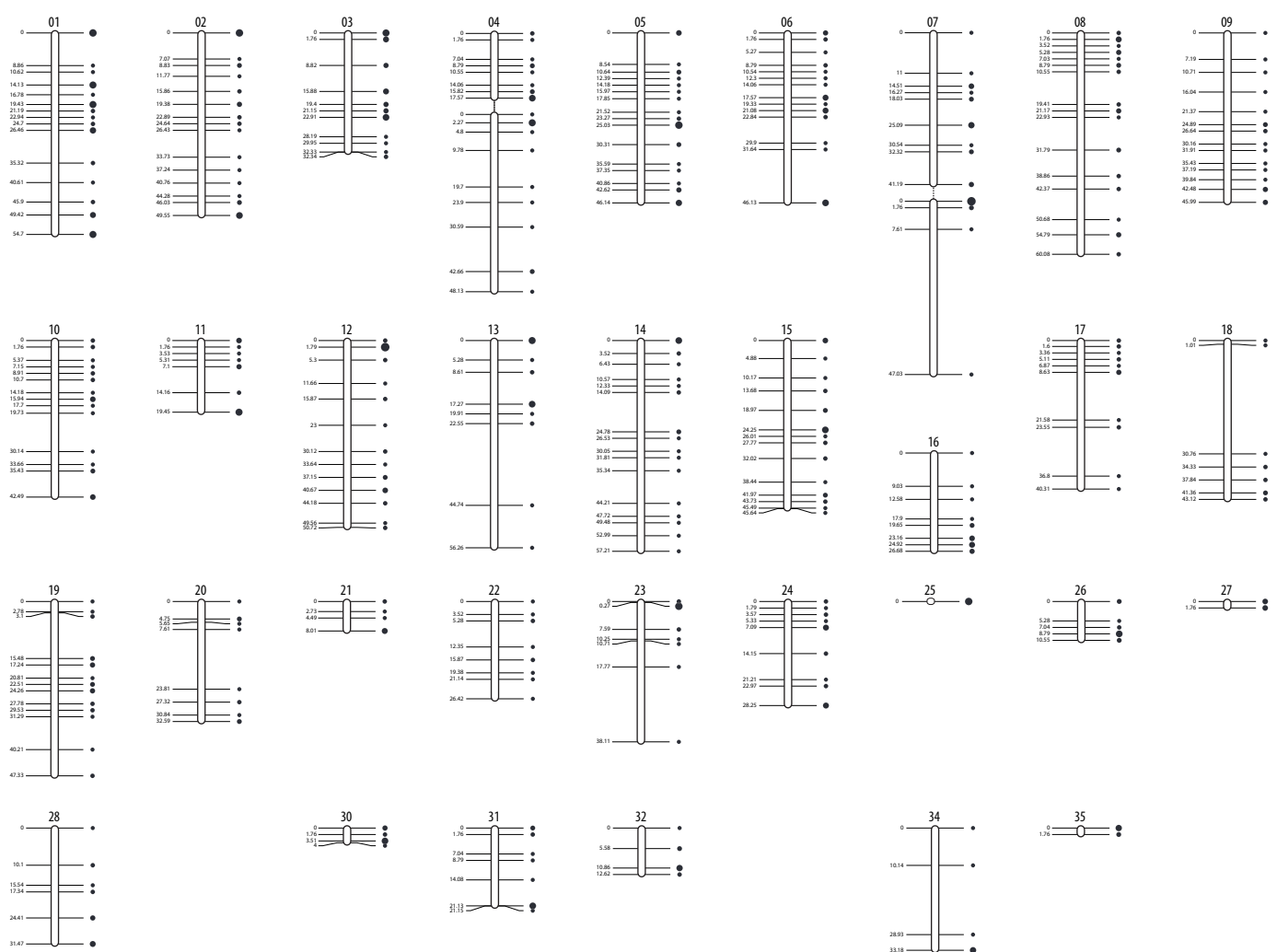

Supplement: Additional file 5: Figure S2. — Female only map of all SNP markers. The positions on the left side of chromosomes are the distance in centi Morgan (cM), Detailed data is provided in Table S2. (PDF 849 kb) [file 12864_2016_2773_MOESM5_ESM.pdf]

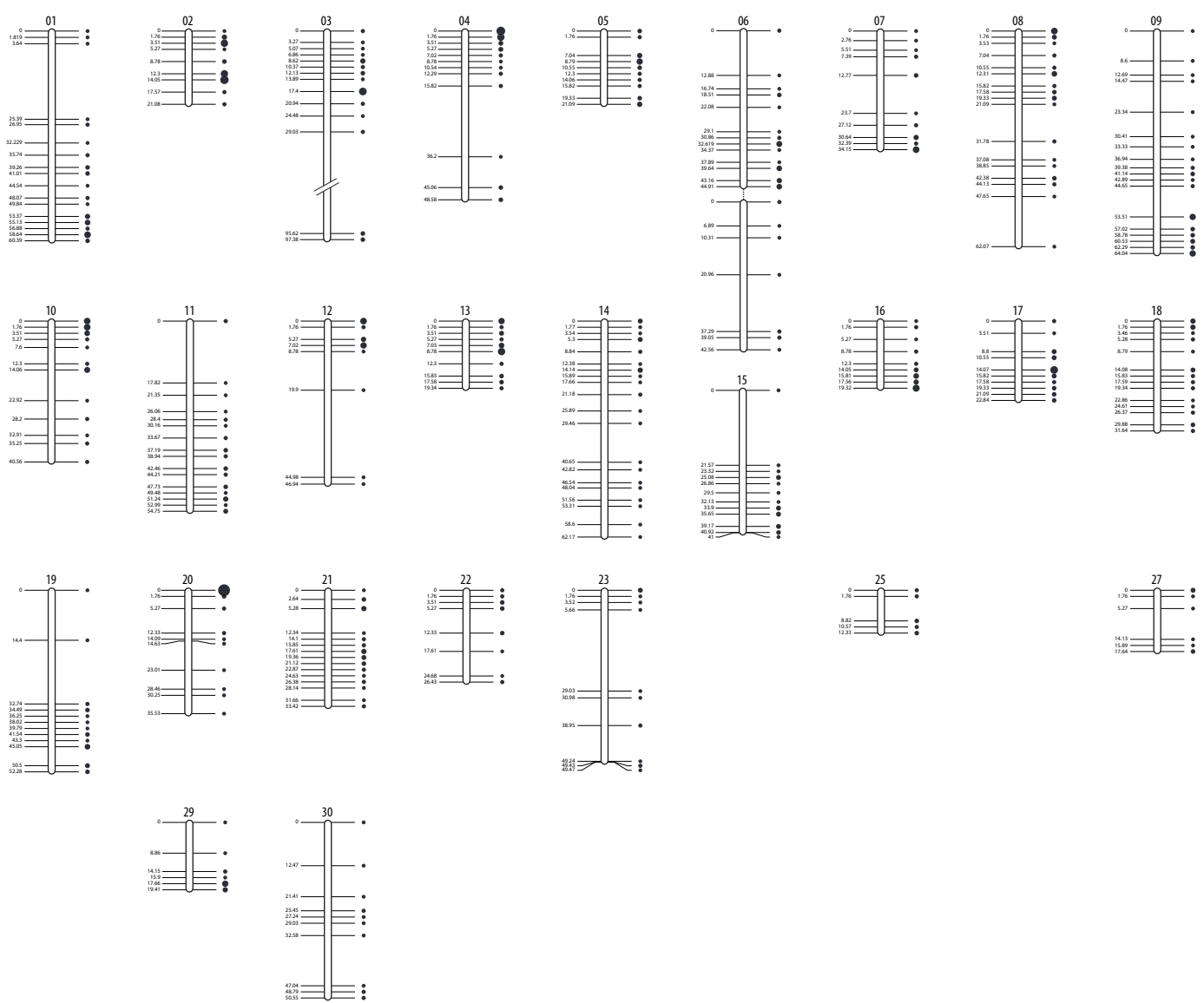

Supplement: Additional file 7: Figure S3. — Male only map of all SNP markers. The positions on the left side of chromosomes are the distance in centi Morgan (cM), Detailed data is provided in Table S2. (PDF 850 kb) [file 12864_2016_2773_MOESM7_ESM.pdf]
