# Supplementary material for: Mapping the sex determination locus in the hāpuku (Polyprion oxygeneios) using ddRAD sequencing
Source: BMC Genomics. 2016 Jun 10;17:448. doi: 10.1186/s12864-016-2773-4 (PMC4902995; doi:10.1186/s12864-016-2773-4)

Figure S4. Examples of SNP assays produced for SphI8298 and SphI6331 in 15 offspring. For each locus two allele-specific PCRs were undertaken with amplicons being separated by electrophoresis (2% agarose gel, 0.5× TAE buffer, containing 100 ng/mL ethidium bromide). Repeat loading of pairs of allele-specific amplicons at 5 minute intervals into the same wells, resolved heterozygous individuals as double-banded phenotypes and homozygous individuals as single-banded phenotypes of different mobility. Direction of migration is indicated by arrows. Phenotypic sex for each sample is given below the banding pattern (M = male, F = female) while allele designations are indicated to the left hand side of the gel image. The single anomalous genotype (SphI1663; heterozygous female,) is identified by *.

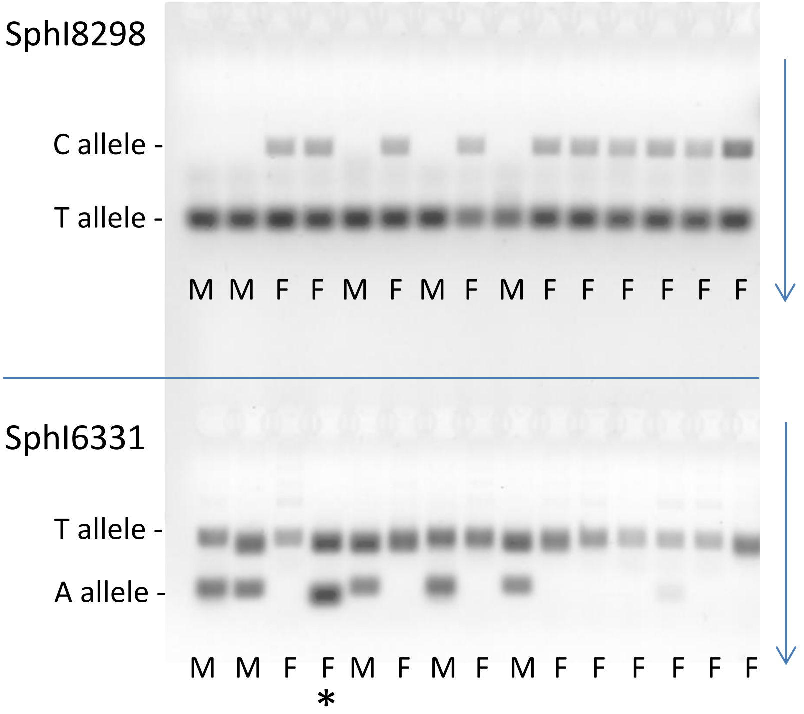

Supplement: Additional file 10: Figure S4. — Gel images of SNP assays for SphI8298 and SphI6331. (PDF 209 kb) [file 12864_2016_2773_MOESM10_ESM.pdf]
